# Clonazepam repurposing in ARID1B patients through conventional RCT and N-of-1 trials: an experimental strategy for orphan disease development

**DOI:** 10.1136/jmg-2024-109951

**Published:** 2024-12-31

**Authors:** Pleuntje J van der Sluijs, Koshar Safai Pour, Cécile L Berends, Matthijs D Kruizinga, Annelieke R Müller, Agnies M van Eeghen, Mar Rodríguez-Girondo, Maria J Juachon, Duco Steenbeek, Adam F Cohen, Rob G J A Zuiker, Gijs W E Santen

**Affiliations:** 1 Department of Clinical Genetics, Leiden University Medical Center, Leiden, the Netherlands; 2 Centre for Human Drug Research, Leiden, the Netherlands; 3 Department of Psychiatry, Leiden University Medical Center, Leiden, the Netherlands; 4 Department of Pediatrics, Haga Teaching Hospital, The Hague, the Netherlands; 5 Department of Pediatrics, Amsterdam University Medical Center, Amsterdam, the Netherlands; 6 Advisium, 's Heeren Loo, Amersfoort, the Netherlands; 7 Department of Medical Statistics and Bioinformatics, Leiden University Medical Center, Leiden, the Netherlands; 8 Department of Rehabilitation Medicine, Maastricht University Medical Center, Maastricht, the Netherlands; 9 Center of Rehabilitation Medicine, Adelante Zorggroep, Maastricht, the Netherlands

**Keywords:** Mental Disorders, Therapeutics

## Abstract

**ABSTRACT:**

**Background:**

Clinical trials for rare disorders have unique challenges due to low prevalence, patient phenotype variability and high expectations. These challenges are highlighted by our study on clonazepam in *ARID1B* patients, a common cause of intellectual disability. Previous studies on Arid1b-haploinsufficient mice showed positive effects of clonazepam on various cognitive aspects.

**Methods:**

This study used a randomised, double-blinded, placebo-controlled, two-way crossover study (RCT), followed by an N-of-1 design. In the crossover study, *ARID1B* patients received clonazepam (max 0.5 mg, two times per day) or a placebo for 22 days with a 3-week washout period. Assessments included safety, tolerability, pharmacokinetics, pharmacodynamics on neurocognitive tasks, behaviour and cognitive function. During phase I of the N-of-1 trial the optimal dosage and individual treatment goals were determined. Phase II evaluated the treatment effect. This phase was composed of three periods: an open-label period with placebo (4 weeks), followed by a double-blinded period (6 weeks), followed by an open-label period in which the patient received clonazepam (4 weeks).

**Results:**

In the clonazepam group (*n*=16, 15 completing both periods), seven (44%) reported improvement on Clinician Global Impression of Improvement versus two (13%) on placebo. 13 (87%) showed ‘no change’ after placebo (two (13%) on clonazepam), while seven (44%) on clonazepam reported deterioration, often linked to side effects (*n*=6), suggesting potential benefit from lower dosing. Three N-of-1 trials with RCT responders saw two patients improve on clonazepam during double-blinding, but clinical evaluation deemed the improvements insufficient.

**Conclusions:**

Our approach shows the feasibility and strength of combining conventional RCT and N-of-1 studies for therapeutic studies in populations with intellectual disabilities, distinguishing real treatment effects from expectation bias. Our findings suggest that clonazepam has no additional therapeutic value in *ARID1B* patients.

**Trial registration number:**

EUCTR2019-003558-98, ISRCTN11225608.

**WHAT IS ALREADY KNOWN ON THIS TOPIC**

- Clinical trials for rare disorders have unique challenges due to the prevalence of the disorder, phenotype variability and (parental) expectations.
- Pathogenic variants in the *ARID1B* gene are among the most frequent causes of intellectual disability.
- Arid1b heterozygous mice show improvement on administration of clonazepam.

**WHAT THIS STUDY ADDS**

- The effect of clonazepam in *ARID1B* patients was investigated using a conventional crossover randomised controlled trial (RCT) design and an N-of-1 design.
- Effects of clonazepam were observed in the RCT, but the N-of-1s only replicated the effect on sleep, suggesting that clonazepam has no additional therapeutic value in *ARID1B* patients.

**HOW THIS STUDY MIGHT AFFECT RESEARCH, PRACTICE OR POLICY**

- This study demonstrates how the N-of-1 trial effectively employs and validates observed effects, differentiating between genuine treatment effects and expectation bias.

## Introduction

*ARID1B* is one of the top mutated genes in heterogeneous intellectual disability, developmental delay and autism cohorts.^1^,^3^ Most patients with Coffin-Siris syndrome (CSS, OMIM 13500)^4^,^7^ have pathogenic variants in *ARID1B*. With the increasing availability and use of genome-wide diagnostics, the number of identified patients has increased. Several hundred cases have been reported in the literature between 2012 and 2022. Almost all *ARID1B* patients have developmental delay,^8^ often with behavioural manifestations such as short attention span and hyperactivity.^8^ In addition, expressive speech is usually severely affected.^8 9^ As a result, individuals with a pathogenic variant in *ARID1B* can face numerous challenges in their daily lives.^8^ These challenges can impact their ability to communicate, socialise or perform daily activities. Unfortunately, until now, no targeted established therapies exist to improve their functioning.

Three Arid1b-haploinsufficient (Arid1b^+/-^) mice models have been generated and investigated.^10^,^13^ These Arid1b^+/-^ mice displayed increased anxiety, and reduced memory, learning and social interaction. Jung *et al* identified a significant reduction in GABAergic inhibitory interneurons in their model, particularly of the parvalbumin-positive subtype.^11^ An investigation of the balance between inhibitory and excitatory synapses revealed a decrease in GABAergic inhibitory synapses, likely causing an inhibition-excitation imbalance. Administering a GABA_A_ receptor positive allosteric modulator, such as clonazepam, may partly reverse this imbalance. Consequently, the investigators administered a single intraperitoneal dose of 0.0625 mg/kg clonazepam to adult mice. Clonazepam-treated Arid1b^+/-^ mice, compared with saline-treated mice, performed better in object recognition, sociability novelty and demonstrated a decrease in anxiety-like behaviour 30–60 min post-treatment, while depression-like behaviour symptoms remained unaffected.^11^

Clonazepam is a Food and Drug Administration (FDA)-approved non-selective benzodiazepine with a half life of 20–40 hours and a Tmax of 1–4 hours after oral dosing. It is currently used to treat seizures in children and adults and occasionally for anxiety. Given its promising effects in the Arid1b^+/-^ mouse model, clonazepam is a potential low-cost treatment suitable for repurposing to improve the functioning of *ARID1B* patients.

In preparation for a randomised controlled clinical trial (RCT) investigating the effects of clonazepam in *ARID1B* patients, we conducted two preliminary studies. One study established the correlation between clonazepam concentrations in plasma and saliva in healthy volunteers, as plasma sampling (via blood draw) is invasive for *ARID1B* patients.^14^ Another study examined the neurocognitive phenotype of 12 *ARID1B* patients and 12 age-matched controls and identified potential biomarkers and outcome measures for treatment evaluation.^15^ Important results of this study were that tests which previously have been evaluated in benzodiazepines,^16 17^ such as for animal fluency, eye movements (saccadic peak velocity and smooth pursuit), body sway, finger tapping and adaptive tracking, could be performed adequately by *ARID1B* patients. Based on these studies, we designed this placebo-controlled study to assess the safety and tolerability, pharmacokinetics (PK) and pharmacodynamics (PD) of clonazepam treatment in *ARID1B* patients.

In the current study, we aim to investigate the effect of clonazepam in *ARID1B* patients and explore biomarkers known to be sensitive to clonazepam with the NeuroCart test procedures. Our research design involves a randomised, double-blinded, placebo-controlled, two-way crossover study, followed by an additional crossover in three patients, resulting in an N-of-1 trial to further delineate the observed treatment effect per patient. Furthermore, by addressing the challenges of validating biomarkers and managing parental expectations, our study may serve as an example of navigating the complexities of research in rare diseases and contribute to improving the care and outcomes of patients with *ARID1B*-related disorders.

## Methods

This study was conducted at the Centre of Human Drug Research (CHDR) in Leiden, the Netherlands, from September 2021 until May 2022. The study was registered at the EU Clinical Trials Register (2019-003558-98). The protocol is both available here and on the International Standard Randomised Controlled Trial Number registry (ISRCTN11225608). The study was conducted according to the Dutch Act on Medical Research Involving Human Subjects, the Dutch codes of conduct regarding medical research with minors and expression of objection by people with mental disabilities and in compliance with Good Clinical Practice.

### Subjects

Inclusion criteria were the identification of a pathogenic variant in *ARID1B* and an age of at least 6 years. Exclusion criteria consisted of a history of severe respiratory problems or severe liver or renal insufficiency, or another medical or psychosocial history making the patient unsuitable for participation as determined by the treating physician or general practitioner.

### Power calculation

Power calculation was performed according to two approaches. (1) Based on the Clinician Global Impression of Improvement (CGI-I) SD of 1.5 points, we calculated that our study would have an 80% power to detect a 1 point difference in CGI-I if 20 patients were included. (2) We also previously calculated the minimally detectable difference for all NeuroCart endpoints with 16 patients (see table 3 in Kruizinga *et al*^15^).

### Study design

#### Randomised, double-blinded, placebo-controlled, two-way crossover study

**Figure 1:**
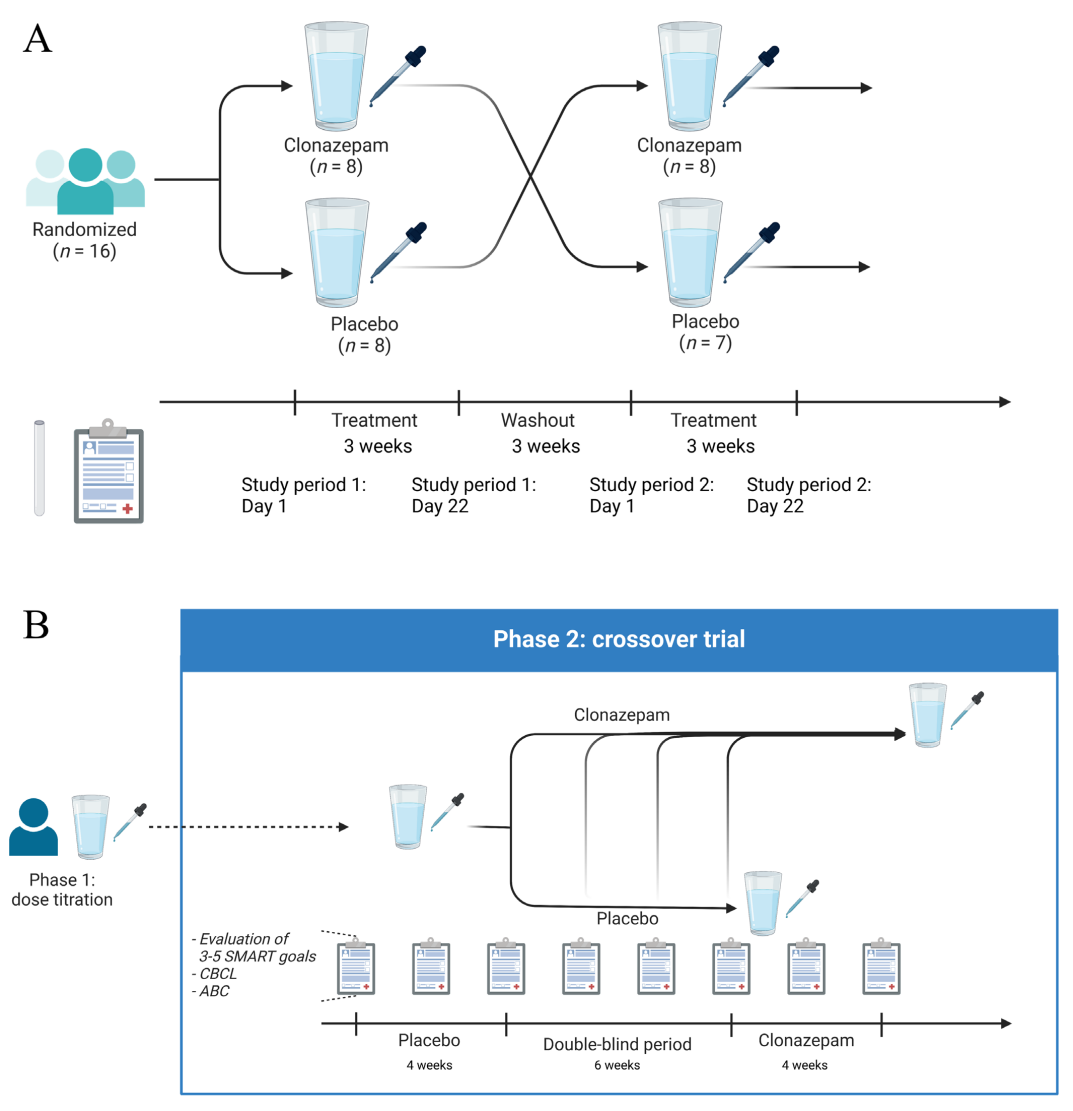
Study design. (A) Conventional crossover randomised controlled trial (RCT). (**B**) Additional crossover study creating an N-of-1 design. ABC, Aberrant Behavior Checklist; CBCL, Child Behavior Checklist; SMART, specific, measurable, achievable, relevant and time-bound. Figures 1A and 1B were created with BioRender.com (2024).

Patients with pathogenic variants in *ARID1B* were recruited via the CSS expertise centre of the Leiden University Medical Center (LUMC), Leiden, the Netherlands. The schedule of assessments is listed in online supplemental table S1. The randomisation code was generated by a study-independent CHDR statistician. In this two-way crossover, placebo-controlled, randomised study, each study period or occasion was 22 days, and a 3-week washout separated each study period or occasion (figure 1A). The starting dose (i.e., 0.01 mg/kg daily) was decided with several physicians with experience in prescribing clonazepam to children, in an attempt to minimise the chance on sedative effects. This dose is half the minimum starting dosage of the administration of clonazepam for anxiety in children (i.e., 0.02 mg/kg daily). Study drug clonazepam or placebo was administered to the subjects as follows: day 1–3: starting dose was 0.005 mg/kg, two times per day (max. 0.5 mg); day 4–6: 0.01 mg/kg, two times per day (max. 0.5 mg); day 7–22: 0.015 mg/kg, two times per day (max. 0.5 mg). Patients received a single starting dose of 0.005 mg/kg (max. 0.5 mg) at their first visit and were subsequently monitored for 5 hours for safety and tolerability, PK and PD effects. If the dose on day 1 was well tolerated, patients took the same dose before bedtime and continued the regimen as described. If side effects related to the treatment were observed, dose was reduced to half of the current dose for 3 days. Thereafter, the original dose at which the side effects first occurred was restarted. If side effects re-emerged, patients remained on the lower dose. In case of clinically significant side effects at the lower dose, patients were excluded.

Furthermore, the clinician and parents determined the Clinician Global Impression of Severity (CGI-S) on day 1 and the CGI-I on day 22.^18^ The teacher or mentor of the subject also assessed the CGI-S and CGI-I. In addition, the parents completed the Aberrant Behavior Checklist (ABC) on day 22.^19^ Within days 1 and 22, patients continued their routine, wore a Steel HR smartwatch and performed home-based assessments (animal fluency, adaptive tracking and finger tapping) two times per week.

#### N-of-1 design

Parents who spontaneously indicated they wanted to continue clonazepam treatment were included in an N-of-1 follow-up protocol. The N-of-1 trials were performed before unblinding and no data of the crossover study were available. This trial was composed of two phases (figure 1B).

##### Phase I: dose titration (variable duration, max. 6 weeks)

The objective of the open-label titration phase was to identify the minimum effective dosage. The open-label titration phase started with a daily dose of 0.01 mg/kg/day in two doses. After 4–7 days, the effects were evaluated and compared with results of the conventional RCT, and the dose was adjusted based on parental feedback, with a maximum dose of 0.03 mg/kg/day. Once parents were satisfied with the effect, this dose was maintained for at least 7 days. After this, the titration phase ended, and the dose was tapered off by 0.01 mg/kg every 3 days. During this phase, individual primary outcome treatment goals were set in consultation with parents using weighed Goal Attainment Scaling (GAS) with six defined levels per goal (see online supplemental table S5)^20 21^ and a paper copy was given to parents.

##### Phase II: a single crossover trial consisting of three treatment periods: placebo, double blind and clonazepam

Phase II lasted 14 weeks and started with an open-label placebo period of 4 weeks. Subsequently, a double-blinded period was started until week 10. During this period, new study medication was started every 2 weeks. This study medication was prepared and provided by our pharmacy. The pharmacist determined randomly when the switch to clonazepam was made during the double-blinded period. This double-blinded period was followed by an open-label period of 4 weeks of clonazepam (figure 1B). There was only one crossover from placebo to clonazepam; therefore, no washout period was required. Regular contact with parents, teacher or mentor by the physician occurred every 2 weeks by phone to evaluate progress in achieving previously established goals. Each goal was scored individually during phase II; from these separate scores, a single aggregated T score was produced using a standardised formula.^22^ Treatment goals were classified in the domains defined by the International Classification of Functioning, Disability and Health-Children and Youth version.^23^ Additionally, parents were asked to complete the Child Behavior Checklist (CBCL) questionnaire every 2 weeks and the ABC questionnaire, as used in the conventional RCT study.

### End of the N-of-1 study

At the end of the study, parents and the study physician were unblinded and decided whether to continue treatment by evaluating their experiences and GAS scores during the study period .

### NeuroCart test procedures

All NeuroCart tests on days 1 and 22 of each study period were conducted according to the protocol by trained instructors. NeuroCart tests were performed as described in online supplemental textbox S1.

### Statistics

#### Randomised controlled trial

All safety and statistical programming were conducted with SAS V.9.4 for Windows or newer (SAS Institute). PK variable programming was conducted with R V.3.6.1 for Windows or newer (R Foundation for Statistical Computing/R Development Core Team, Vienna, Austria, 2010). To establish treatment effects, the repeatedly measured crossover PD endpoints were analysed by mixed model analyses of variance with treatment, period, time and treatment by time as fixed effects, with subject, subject by treatment and subject by time as a random effect, and with the (average) baseline value as covariate.

#### N-of-1 design

All analyses were performed using SPSS V.29. For the primary outcome, linear mixed models (LMM) were used to compare GAS-T scores between placebo and clonazepam treatment, blinding was added as a fixed covariate. The individual was included as a random intercept. In a similar manner, LMM was used to determine whether CBCL and ABC questionnaire results were different between placebo and clonazepam treatment.

## Results

### Randomised controlled trial

#### Patients

**Figure 2:**
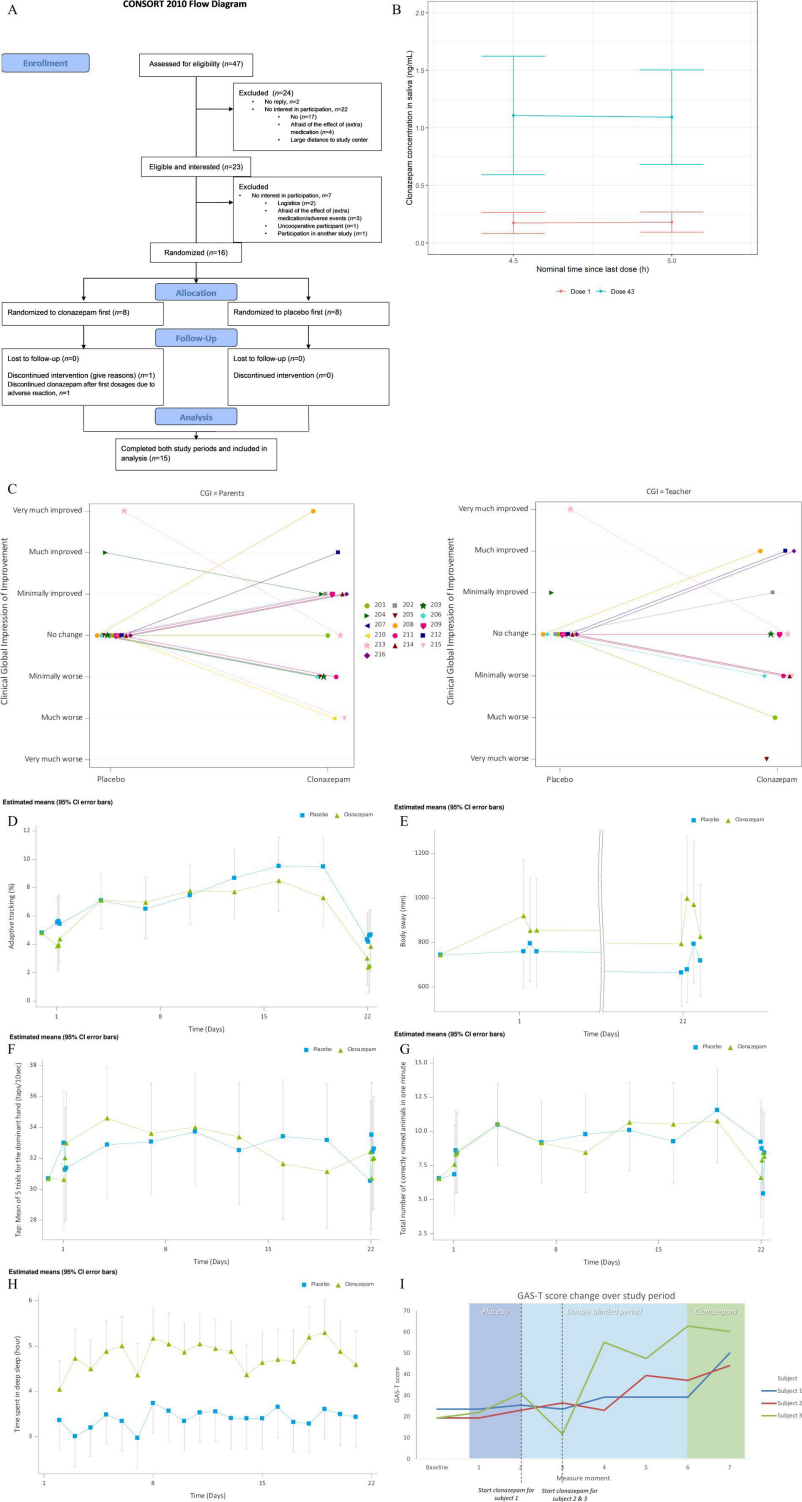
Randomised controlled trial (RCT): (A) Consolidated Standards of Reporting Trials (CONSORT) flow diagram; (**B**) individual concentrations of clonazepam concentration in saliva (ng/mL)—coloured per dose; (**C**) Clinician Global Impression of Improvement (CGI-I); (**D**) adaptive tracking; (**E**) body sway; (**F**) tapping; (**G**) animal fluency; (**H**) deep sleep; (**I**) N-of-1: Goal attainment scaling (GAS)-T change. CFB, Change From average Baseline.

47 parents of patients (figure 2A) with a pathogenic variant in *ARID1B* were approached between May 2021 and January 2022, of which 16 consented to participate with their child (table 1 and online supplemental table S2). The pathogenic *ARID1B* variants identified in these 16 patients are all variants predicted to lead to haploinsufficiency. Of these 16 patients, 11 (69%) were female and the age varied between 6 and 33 years (median: 17). All but one patient had an intellectual disability, one patient had an IQ score within the normal range, but was diagnosed with developmental delays in combination with recurrent otitis media and laryngomalacia. These 16 patients were randomly assigned to receive either clonazepam or placebo, of which 15 completed both study periods. One subject stopped at day 4 of the clonazepam treatment period due to side effects consistent with a paradoxical reaction to clonazepam.

**Table 1:** Demographics

Sex	Weight (kg)	Height (cm)	BMI (kg/m^2^)	CGI-S
Female	65.0	154.0	27.4	Slightly ill
Female	60.5	164.0	22.5	Slightly ill
Male	57.1	164.9	21.0	Minimal ill
Female	55.4	147.7	25.4	Minimal ill
Female	24.0	125.8	15.2	Slightly ill
Female	51.0	150.2	22.6	Slightly ill
Female	28.0	131.6	15.9	Slightly ill
Male	39.0	157.9	15.7	Slightly ill
Female	50.0	152.7	21.2	Minimal ill
Male	36.0	121.7	24.1	Slightly ill
Female	38.6	139.3	19.9	Not ill
Female	68.5	160.8	26.5	Minimal ill
Female	19.1	115.0	14.4	Moderately ill
Female	55.0	167.0	19.0	Slightly ill
Male	74.0	179.5	22.2	Not ill
Male	54.0	170.0	18.7	Moderately ill
Female=11	Avg=52.50	Avg=153.4	Avg=21.12	

AvgaverageBMIbody mass indexCGI-S, Clinical Global Impression of Severity

#### PK assessment

Online supplemental table S3 shows the administered clonazepam dosages per subject. Figure 2B shows the individual clonazepam concentrations in saliva. After the first day of dosing, the drug’s mean (SD) concentration was 0.17 ng/mL and 0.18 ng/mL (0.09) at 4.5 and 5 hours post-dose, respectively. On day 22, the mean (SD) concentration of the last dose was 1.11 ng/mL (0.52) and 1.10 ng/mL (0.41) at 4.5 and 5 hours post-dose, respectively. The accumulation ratio observed for the patients was approximately 6.1–6.5, indicating significant drug accumulation over time.

##### Titration adjustments of clonazepam

During the study, dosage adjustments necessitated by side effects were made for five out of 15 patients while taking clonazepam. These adjustments, consistently involving a halving of the dose, were executed on day 6, 7 or 8. In three patients, the dose was not increased to the prior dosing at the parents’ request on day 11. No dosage modifications were required for patients while they were on the placebo.

### PD assessment

#### Clinical Global Impression of Improvement

The mean and individual CGI-I values reported by parents and teachers are shown in figure 2C.

#### CGI-I interviews with parents

Based on CGI-I interviews with parents, out of 15 patients who received a placebo treatment, for 13 patients (87%) parents reported no change, for two patients (13%) parents reported improvement and none reported worsening (online supplemental table S4).

Out of 15 patients who completed clonazepam treatment, two patients (13%) reported no change, seven patients (47%) reported an improvement and six patients (40%) reported worsening in the clonazepam treatment period compared with day 1 pre-dose. Examples of self or parent-reported improvement in the clonazepam group included increased expressive speech (*n*=4), self-reported calmness (*n*=3), experiencing fewer emotional outbursts and/or more susceptible to reason (*n*=2), better concentration (*n*=2), taking more initiative (*n*=1), better at indicating own boundaries (*n*=1) and better sleep (*n*=5). Self-reported or caregiver-reported worsening was linked to adverse events in all cases except for one case (see table 3 and the paragraph ‘adverse events’). In this patient, the reported worsening involved an increased preoccupation with herself, more frequent outbursts of emotion directed at her surroundings and greater difficulty engaging with others.

#### CGI-I interviews with teacher or mentor

In most cases, the CGI-I scores based on interviews with teacher or mentor overlapped with the evaluation with parents. In four cases, CGI-I from both treatment periods was not available. Based on the CGI-I with the teacher or mentor, four patients improved, five patients worsened, and in two patients, no change was observed in the clonazepam treatment period.

#### Aberrant Behavior Checklist

Another self-reported outcome was the ABC which was separately completed by the parents and teacher or mentor (table 2). The parental ABC indicated a significant increase in hyperactivity score during the clonazepam compared with the placebo treatment period after adjusting for baseline measures.

**Table 2:** NeuroCart results, Steel HR sleep and ABC questionnaire

			Least squares mean	
		Treatment P value	Placebo (*n=*15)	Clonazepam (*n=*15)
NeuroCart	Smooth pursuit	0.64	12.65	12.36
	Body sway	0.16	737.36	885.98
	Tap: mean of 5 trials for the dominant hand (taps/10 s)	0.92	32.58	32.40
	Adaptive tracking (%)	0.10	6.40	5.34
	Animal fluency	0.96	8.9	8.9
Withings Steel HR	Time spent in light sleep (hour)	<0.01	5.10	4.14
	Time spent in deep sleep (hour)	<0.01	3.41	4.79
	Time spent waking up (hour)	0.49	0.05	0.04
	Time spent in total sleep	0.06	8.49	8.92
	Number of times waking up during sleep (count)	<0.01	4.1	2.3
ABC parents	1: Irritability	0.95	6.2	6.3
	2: Lethargy	0.84	11.4	12.1
	3: Stereotypy	0.44	1.6	2.5
	4: Hyperactivity	0.05	7.9	14.3
	5: Inappropriate speech	0.73	1.3	1.4

ABC, Aberrant Behavior Checklist

#### NeuroCart and Withings Steel HR

No differences between the clonazepam and placebo period were observed on the NeuroCart test (table 2, figure 2D–H). Saccadic eye movement measurements were successful for only two patients on both study periods’ first and last days. Therefore, no analyses were performed on these data. The Steel HR watch measurements showed that compared with the placebo period, patients during the clonazepam period spent more time in deep sleep and less time in light sleep. However, their total sleep time was comparable between study periods (table 2).

### Safety and tolerability/adverse events

In total, for nine patients (56%), more than one adverse event was reported (table 3) while taking clonazepam, compared with six patients (38%) taking a placebo. During the clonazepam treatment, fatigue was the most frequently reported adverse event (*n*=8), followed by somnolence (*n=*2). For five of the six patients with a worsening CGI-I score, adverse events linked to clonazepam were reported (fatigue, *n*=5; nausea, *n*=1). A paradoxical drug reaction was observed in one patient on day 4 during clonazepam treatment. After the first increase in dosing on day 4, this patient displayed agitated behaviour at home and school, restlessness and sleepiness. In consultation with the parents, it was decided to exclude this patient from the study early.

**Table 3:** Summary of number of subjects in the RCT with TEAEs by treatment, SOC, PT and severity

System Organ Class/Preferred Term	Clonazepam (*n=*16)			Placebo (*n=*15)		
	Mild	Moderate	Severe	Mild	Moderate	Severe
Any events	10	2	–	7	–	–
Gastrointestinal disorders						
Upper abdominal pain	1	1	–	–	–	–
Nausea	–	–	–	1	–	–
General disorders and administration site conditions						
Fatigue	8	–	–	2	–	–
Feeling drunk	1	–	–	–	–	–
Paradoxical drug reaction	–	1	–	–	–	–
Infections and infestations						
Coronavirus infection	1	–	–	–	–	–
Nasopharyngitis	1	–	–	1	–	–
Injury, poisoning and procedural complications						
Head injury	1	–	–	–	–	–
Musculoskeletal and connective tissue disorders						
Bursitis	1	–	–	–	–	–
Nervous system disorders						
Balance disorder	2	–	–	–	–	–
Disturbance in attention	1	–	–	–	–	–
Dysarthria	1	–	–	–	–	–
Headache	–	–	–	–	–	–
Slow response to stimuli	1	–	–	–	–	–
Somnolence	1	–	–	–	–	–
Psychiatric disorders	1			2		
Bradyphrenia		–	–	1	–	–
Disinhibition	1	–	–	–	–	–
Impulse control disorder	1	–	–	–	–	–
Inappropriate affect		–	–	1	–	–
Respiratory, thoracic and mediastinal disorders	2	–	–	–	–	–
Oropharyngeal pain	1	–	–	–	–	–
Rhinorrhoea	1	–	–	–	–	–

PT, Preferred TermRCTrandomised controlled trialSOC, System Organ Class; TEAE, treatment-emergent adverse event

There was no observed dose-dependent treatment effect for any of the parameters, and there were no differences in treatment effects based on phenotype severity, sex or age.

### N-of-1

Three parents of the 15 patients expressed interest in continuing clonazepam treatment after completing the trial (see online supplemental table S6).

#### Phase I

Several patients or parents reported a sedative effect of clonazepam. Therefore, to identify the minimal effective dosage of clonazepam, we added a titration phase I in the N-of-1 design. The optimal dose at the end of the open-label titration phase I differed from the dose used during the RCT for all patients. Online supplemental table S7 provides an overview of the domains for which specific goals were defined.

#### Phase II

All three enrolled patients completed this phase and their data were analysed. Online supplemental table S8 and figure 2I show the change in the GAS-T score during phase 2. While all patients exhibited improvement on the GAS-T score, only subject 3 showed a distinct improvement in the GAS-T score, specifically on switching from placebo to clonazepam during the double-blinded period. Further exploration of the indicated effect using the GAS scoring and parental feedback indicated an effect on sleep in two patients and a small effect on behaviour in one patient. No relevant changes at the individual level or group level were measured using the behaviour questionnaires ABC and CBCL (see online supplemental table S9), and no adverse events, side effects or paradoxical reactions were reported.

#### Treatment evaluation and informed decision-making

At the end of phase II, the results were discussed with the parents of patient 1 and her physician in intellectual disability medicine. The parents expressed the need to take some time to consider the results, and care was transferred to the patient’s own physician, who was also present during the evaluation. For patient 2, who demonstrated an observed effect only on sleep, the parents decided to discontinue clonazepam treatment. Similarly, for subject 3, who showed an effect on sleep and a potentially small effect on behaviour, the parents, together with their child’s physician, concluded that clonazepam did not significantly improve the child’s functioning and chose to discontinue its use.

## Discussion

This randomised, double-blinded, placebo-controlled crossover study, followed by N-of-1 design, is the first to investigate the effects of clonazepam in patients with an *ARID1B*-related intellectual disability. In our studies, aside from the effect on sleep observed by parents, no significant improvements in functioning were observed for the clonazepam group compared with the placebo group. A large heterogeneity in treatment response was present, with nearly half of the parents and patients themselves reporting notable improvement, and nearly half reporting a worsening in function. The reported improvements were further investigated in a subset of our population using an N-of-1 study. Th results of this study confirmed the effect on sleep and showed no significant improvements on the other domains, illustrating the difficulties when assessing drug effects without objective clinical endpoints and indirectly through parents/caregivers in this highly variable population.

Ultimately, 16 patients started the study, and 15 patients completed the full study. Therefore, the power to detect a 1 point difference on the CGI-I was not 80% (at *n*=20) but 67% (at *n*=15). Given the complete lack of signal and the subsequent results from the N-of-1 trials we consider it unlikely that including five additional patients would have led to a different conclusion.

The titration phase of the N-of-1 trials resulted in an observed effect with slightly lower dosages for all three patients compared with the dose they received during the crossover study. We consider it unlikely that the lack of effect on other domains, except sleep, in the N-of-1 trials can be explained by the lower dosage in these studies. Since the parents did observe an effect of clonazepam in phase I, and a clear effect of clonazepam on sleep, similar to the crossover study, was measured in phase II. We therefore consider it unlikely that a higher dose would have resulted in better efficacy.

Previous research in an Arid1b^+/-^ mouse model showed promising results for clonazepam compared with saline treatment.^11^ In the Arid1b^+/-^ mouse model, clonazepam improves novel object recognition, social novelty and anxiety.^11^ Although it is always critical to approach extrapolation from mouse models to human patients with caution, the observed improvements in the mouse model suggested potential overlap with positive effects reported in patients, such as improved concentration, emotion regulation and calmness. However, in this trial, these encouraging results from the mouse model did not translate into positive outcomes for humans.

### CGI and ABC

Variable treatment effects of clonazepam were observed in the *ARID1B* patient population based on the parental CGI-I results, with seven patients improving, six worsening and two showing no change. This variability may be attributed to population heterogeneity,^8^ but could also be explained by random variations or parents who are highly motivated to observe drug effects. Reported side effects, such as fatigue and somnolence, align with clonazepam’s known central nervous system effects and likely contributed to the reported worsening in the functioning of a subset of patients. Lowering the dose may have improved the benefit-risk ratio for this group.

An unexpected finding was that parents reported a higher hyperactivity domain score on the ABC questionnaire during the clonazepam period compared with placebo (borderline statistically significant). This domain, known as ‘Hyperactivity/Noncompliance’, measures levels of hyperactivity and disobedience. Given clonazepam’s sedative properties, it would be expected that patients’ activity levels during clonazepam treatment would be lower or similar to those during placebo treatment. However, the observed agitation and restlessness in one patient may have been present in a milder form in more patients. We speculate that clonazepam more frequently displays paradoxical effects in patients with *ARID1B*-related intellectual disability.

### NeuroCart

Contrary to what we had anticipated, we could not show an improved performance for clonazepam compared with placebo treatment on NeuroCart tests.^16 17^ The reasons behind the lack of a significant diminished effect remain uncertain. The most likely explanation is that clonazepam has no effect on these tests. Alternatively, the side effect of clonazepam could offset the expected improvement in psychomotor tests. The possibility that this patient population was unable to perform the test accurately could be another consideration, though this seems unlikely given that prior research has shown that these tests could be administered to this patient group.^15^

### Goal attainment scaling

We used GAS in our N-of-1 design to evaluate individual treatment effects and offer personalised assessments. GAS is sensitive to change, and its patient-centred focus increases relevancy and the likelihood of detecting relevant differences in treatment outcomes. However, it is important to consider the subjectivity of goal setting and rating, the lack of standardisation, difficulty of choosing goals that are specific, measurable, achievable, relevant and time-bound, and limited generalisability. As demonstrated in our N-of-1 design, although GAS provides meaningful and personalised outcomes, the potential bias towards scoring progress^24^ should be interpreted cautiously, considering the subjective nature of the defined goals and the need to discuss the clinical significance of the measured differences with parents or caregivers. Additionally, in the evaluation process, it is important to assess the overall effect of the medication and consider the possibility of treatment effects not captured by the predefined goals.^24^

### Strengths and limitations

This is the first clinical study for *ARID1B* and/or CSS. Strengths of this study include the use of the innovative trial design combining a crossover design that allows each patient to serve as their control, with an optional additional placebo-controlled RCT, resulting in an N-of-1 design, a sizeable cohort, a low dropout rate, repeated measurement design and the involvement of parents in the study design.

Our study also has several limitations. Only patients able to handle multiple testing on the study days were included. This may have introduced a selection bias towards less affected patients. An additional issue complicating determination of a clinically meaningful change is the reported placebo response of 20% in autism clinical trials.^25^ This report indicates that studies involving children and adolescents, employing caregiver ratings and ensuring minimal biases and allocation concealment risks were associated with the highest risk of placebo response. In addition, caregiver or self-reported measures may introduce expectation bias. This may have led to an accentuation of this placebo effect by overshadowing subtle changes that may happen in the treatment period.^26^ We tried to limit the expectation bias by using a double-blinded design of both studies, and the low number of patients that improved during the placebo period of the RCT makes it probable that these effects were minimal in this study. On the other hand, the results of the N-of-1 study of subject 1 (figure 2I) clearly demonstrate the presence of expectation bias during the last unblinded period. Another potential limitation of this study is that the endpoints were very general. We chose CGI since it was unknown what kind of effect clonazepam might have in *ARID1B* patients, which complicates the choice of appropriate, specific study endpoints. On the other hand, these broad CGI-I evaluations made it possible to catch any potential effect that may have been missed with a more specific endpoint and enabled us to estimate the treatment effect of clonazepam compared with placebo. Selection bias may be present in the selection of patients for our N-of-1 trial. Only patients whose parents approached the study team about the continuation of clonazepam treatment were included, creating a bias towards patients with a potential positive effect of clonazepam during the crossover study. However, we would expect this bias to be in favour of clonazepam effect. Furthermore, we cannot exclude that the dose titration phase of the N-of-1 might have resulted in patients or the parents or caretaker recognising the effects of clonazepam in the double-blinded phase II. However, we felt this disadvantage was outweighed by the positive effects of allowing a thorough determination of the optimal dose, and given the results it is unlikely that this effect changed the outcome.

### Conventional randomised clinical trial and N-of-1 studies

The conventional/parallel-group RCTs and the N-of-1 design are distinct but complementary approaches in clinical research, each with unique strengths. RCTs provide a rigorous framework, minimising biases and allowing for unbiased assessment of treatment efficacy on a larger scale. They offer more objective results through randomisation and blinding. However, RCTs may not capture individual variations and personalised responses to interventions.

On the other hand, the N-of-1 design offers a personalised and patient-centred approach, monitoring individual responses to treatments over time. They capture nuances and fluctuations in symptoms, providing valuable insights at the individual level. However, the smaller sample size in N-of-1 studies and subjective endpoints may increase the risk of type 1 errors.^24 27^

To confirm clonazepam’s effects in individual patients and limit expectation bias, our study combined a conventional RCT with a crossover design, followed by an additional crossover in three patients, resulting in an N-of-1 trial. This approach maximised the strengths of both methods while considering time and cost constraints.

In our case, the N-of-1 trials further explored observed effects from the RCT. Alternatively, starting with N-of-1 initially could have allowed for a detailed understanding of intervention effects and informed the design of a more targeted RCT with specific endpoints.

## Conclusions

Altogether, the results of our RCT and following N-of-1 trials suggest that clonazepam has no effect on *ARID1B* patients. Some effects were observed in a subset (*n*=6) of patients in the RCT. The N-of-1s in three of these patients only replicated the effect on sleep. Our study may serve as an example of navigating the complexities of research in rare diseases and contribute to improving the care and outcomes of patients with *ARID1B*-related disorders by addressing the challenges of validating biomarkers and identifying parental expectation bias. We demonstrated how the N-of-1 trial is effectively employed to explore and validate observed effects, enabling us to differentiate between genuine treatment effects and expectation bias.

## supplementary material

10.1136/jmg-2024-109951 online supplemental file 1

## Data Availability

Data are available in a public, open access repository. Data are available upon reasonable request.

## References

[1] Hoyer J, Ekici AB, Endele S (2012). Haploinsufficiency of ARID1B, a member of the SWI/SNF-a chromatin-remodeling complex, is a frequent cause of intellectual disability. Am J Hum Genet.

[2] Deciphering Developmental Disorders S (2017). Prevalence and architecture of de novo mutations in developmental disorders. Nature New Biol.

[3] Gillentine MA, Wang T, Eichler EE (2022). Estimating the Prevalence of De Novo Monogenic Neurodevelopmental Disorders from Large Cohort Studies. Biomedicines.

[4] Tsurusaki Y, Okamoto N, Ohashi H (2012). Mutations affecting components of the SWI/SNF complex cause Coffin-Siris syndrome. Nat Genet.

[5] Santen GWE, Aten E, Vulto-van Silfhout AT (2013). Coffin-Siris syndrome and the BAF complex: genotype-phenotype study in 63 patients. Hum Mutat.

[6] Wieczorek D, Bögershausen N, Beleggia F (2013). A comprehensive molecular study on Coffin-Siris and Nicolaides-Baraitser syndromes identifies a broad molecular and clinical spectrum converging on altered chromatin remodeling. Hum Mol Genet.

[7] Chen C-A, Lattier J, Zhu W (2022). Retrospective analysis of a clinical exome sequencing cohort reveals the mutational spectrum and identifies candidate disease-associated loci for BAFopathies. Genet Med.

[8] van der Sluijs PJ, Jansen S, Vergano SA (2019). The ARID1B spectrum in 143 patients: from nonsyndromic intellectual disability to Coffin-Siris syndrome. Genet Med.

[9] Santen GWE, Aten E, Sun Y (2012). Mutations in SWI/SNF chromatin remodeling complex gene ARID1B cause Coffin-Siris syndrome. Nat Genet.

[10] Celen C, Chuang J-C, Luo X (2017). *Arid1b* haploinsufficient mice reveal neuropsychiatric phenotypes and reversible causes of growth impairment. Elife.

[11] Jung E-M, Moffat JJ, Liu J (2017). Arid1b haploinsufficiency disrupts cortical interneuron development and mouse behavior. Nat Neurosci.

[12] Shibutani M, Horii T, Shoji H (2017). Arid1b Haploinsufficiency Causes Abnormal Brain Gene Expression and Autism-Related Behaviors in Mice. Int J Mol Sci.

[13] Moffat JJ, Jung E-M, Ka M (2019). The role of ARID1B, a BAF chromatin remodeling complex subunit, in neural development and behavior. Prog Neuropsychopharmacol Biol Psychiatry.

[14] Kruizinga MD, Zuiker RGJA, Bergmann KR (2022). Population pharmacokinetics of clonazepam in saliva and plasma: Steps towards noninvasive pharmacokinetic studies in vulnerable populations. Br J Clin Pharmacol.

[15] Kruizinga MD, Zuiker RGJA, Sali E (2020). Finding Suitable Clinical Endpoints for a Potential Treatment of a Rare Genetic Disease: the Case of ARID1B. Neurotherapeutics.

[16] Zuiker RGJA, Chen X, Østerberg O (2016). NS11821, a partial subtype-selective GABAA agonist, elicits selective effects on the central nervous system in randomized controlled trial with healthy subjects. *J Psychopharmacol*.

[17] Groeneveld GJ, Hay JL, Van Gerven JM (2016). Measuring blood-brain barrier penetration using the NeuroCart, a CNS test battery. Drug Discov Today Technol.

[18] Guy W (1976). Clinical global impression. Assess man for Psychopharmacol.

[19] Aman MG, Singh NN, Stewart AW (1985). The aberrant behavior checklist: a behavior rating scale for the assessment of treatment effects. Am J Ment Defic.

[20] Kiresuk TJ, Sherman RE (1968). Goal attainment scaling: A general method for evaluating comprehensive community mental health programs. Community Ment Health J.

[21] Dekkers K, Viet E, Eilander H https://revant.nl/dynamic/media/1/documents/Gas/handleiding_GAS.pdf.

[22] Kiresuk TJ, Smith A, Cardillo JE (1994). Goal attainment scaling: applications, theory, and measurement. L. Erlbaum Associates.

[23] World Health Organization (2007). International classification of functioning, disability and health: children & youth version: ICF-CY.

[24] Steenbeek D, Ketelaar M, Galama K (2007). Goal attainment scaling in paediatric rehabilitation: a critical review of the literature. Dev Med Child Neurol.

[25] Siafis S, Çıray O, Schneider-Thoma J (2020). Placebo response in pharmacological and dietary supplement trials of autism spectrum disorder (ASD): systematic review and meta-regression analysis. Mol Autism.

[26] McConachie H, Parr JR, Glod M (2015). Systematic review of tools to measure outcomes for young children with autism spectrum disorder. Health Technol Assess.

[27] Urach S, Gaasterland C, Posch M (2019). Statistical analysis of Goal Attainment Scaling endpoints in randomised trials. Stat Methods Med Res.

