# Supplementary material for: Clonazepam repurposing in ARID1B patients through conventional RCT and N-of-1 trials: an experimental strategy for orphan disease development
Source: J Med Genet. 2024 Dec 31;62(3):e109951. doi: 10.1136/jmg-2024-109951 (PMC11877031; doi:10.1136/jmg-2024-109951)
Supplement: online supplemental file 1 [file jmg-62-3-s001.pdf]

SUPPLEMENTARY MATERIAL

Clonazepam repurposing in *ARID1B* patients through conventional RCT and N-of-1 Trials - An experimental strategy for orphan disease development

Pleuntje J. van der Sluijs*[1], Koshar Safai Pour*[2,3], Cécile L. Berends[2], Matthijs D. Kruizinga[2,4,5], Annelieke R. Müller[6,7], Agnies M. van Eeghen[6,7], Mar Rodríguez Girondo[8], Maria J. Juachon[2], Duco Steenbeek[9], Adam F. Cohen[2], Rob G.J.A. Zuiker[*2], Gijs W.E. Santen[*1]

1. Department of Clinical Genetics, Leiden University Medical Center, the Netherlands.
2. Centre for Human Drug Research, Leiden, the Netherlands.
3. Department of Psychiatry, Leiden University Medical Center, the Netherlands.
4. Department of Pediatrics, Juliana Children's Hospital – Haga teaching hospital, the Hague, the Netherlands
5. Department of Pediatrics, Leiden University Medical Centre, Leiden, The Netherlands
6. Advisium, 's Heeren Loo, Amersfoort, the Netherlands.
7. Department of Pediatrics, Emma Children's Hospital, Amsterdam University Medical Center, Amsterdam, The Netherlands.
8. Department of Medical Statistics and Bioinformatics, Leiden University Medical Center, Leiden, The Netherlands.
9. Department of Rehabilitation Medicine, Maastricht University Medical Center / Adelante Rehabilitation, Maastricht, The Netherlands.

* Shared first and last author

Correspondence to

Gijs Santen, MD, PhD
E-mail: santen@lumc.nl

Contents

Textbox S1: NeuroCart Test procedures 3
Table S1: *ARID1B* variants of included patients .... **Error! Bookmark not defined.**
Table S2. Schedule of assessment .... **Error! Bookmark not defined.**
Table S3: Self-reported improvements reported during CGI-I interview 8
Table S4: Example of a SMART-defined goal using Goal Attainment Scaling 9
Table S5: N-of-1: Patient characteristics 10
Table S6: Treatment goals (ICF) 11
Table S7: Primary outcome 12
Table S8: Behavioral questionnaires in the *N*=1 study 13

2

Textbox S1: NeuroCart Test procedures

All NeuroCart tests on day 1 and 22 of each study period were conducted according to the protocol by trained instructors. Description of the NeuroCart test procedures can be found in Table 1.

Cognition

For the animal fluency test, subjects were asked to verbally produce as many different animals as they could sum up within 60 seconds[1]. Only unique animals (excluding species) were counted, and juvenile and adults were counted as one.

Eye Tracking

Recording of eye movements was performed in a quiet room with dimmed illumination. Analysis was conducted with a microcomputer-based system for a sampling of eye movements. Disposable electrodes (Ambu Blue Sensor N) were applied on the forehead and beside the lateral canthi of both eyes. Skin resistance was minimalized before measurements. Head movements were restrained using a fixed head support. Subjects were asked to focus on a moving dot displayed on a computer screen. Saccadic eye movements were recorded for stimulus amplitudes of approximately 15° to either side. Fifteen saccades were recorded with inter-stimulus intervals varying randomly between 3 and 6 s. Average values of saccadic peak velocity (degrees/s) of correct saccades were recorded. At least five detected saccades were necessary to include for statistical analysis. For smooth pursuit eye movements, the target moves sinusoidally at frequencies ranging from 0.3 to 1.1 Hz. Four cycles were recorded for each stimulus frequency. The time the eyes were in smooth pursuit of the target was calculated and expressed as a percentage of stimulus duration[2].

Executive Functioning Assessments

The adaptive tracking test is a pursuit-tracking task and was performed as described by Borland and Nicholson using customized equipment and software[3]. The subjects were instructed to keep a dot inside a moving circle by operating a joystick. The speed of the moving circle is adapted in response to the subject's performance. After a run-in period of 30 seconds, the average tracking performance (%) of 3.5 minutes was used for statistical analysis. Body sway was conducted by all subjects and assessed using a pot string meter (Celesco) based on a Wright ataxiameter, with a string attached to the waist[4]. All body movements over 2 minutes were integrated and expressed as sway in mm. Before starting a measurement, subjects were asked to stand still and comfortable with their hands in a relaxed position. Subjects wore an eye cap to block sight. The finger tapping test was performed and adapted from the Halstead Reitan Test Battery[5]. The speed of finger tapping was measured for the index finger for the dominant hand; a session contained five performances of 10s. Subjects were instructed to tap a button with the dominant hand's index finger as quickly as possible. The mean tapping rate was used for statistical analysis.

Trial@home

During the 20 days between the in-house period, subjects wore a Steel HR watch (Withings, Issy-les-Moulineaux, France), incorporated in the CHDR MORE trial@home platform and registered several accelerometer-derived sleep parameters. The subjects were also instructed to perform twice-weekly finger tapping, adaptive tracking and animal fluency at home.

3

References:
1. Tombaugh TN, Kozak J, Rees L. Normative data stratified by age and education for two measures of verbal fluency: FAS and animal naming. *Arch Clin Neuropsychol*. Feb 1999;14(2):167-77.
2. van Steveninck AL, Schoemaker HC, Pieters MS, Kroon R, Breimer DD, Cohen AF. A comparison of the sensitivities of adaptive tracking, eye movement analysis and visual analog lines to the effects of incremental doses of temazepam in healthy volunteers. *Clin Pharmacol Ther*. Aug 1991;50(2):172-80. doi:10.1038/clpt.1991.122
3. Borland RG, Nicholson AN. Visual motor co-ordination and dynamic visual acuity. *Br J Clin Pharmacol*. 1984;18 Suppl 1(Suppl 1):69S-72S. doi:10.1111/j.1365-2125.1984.tb02583.x
4. van Steveninck AL, Gieschke R, Schoemaker HC, et al. Pharmacodynamic interactions of diazepam and intravenous alcohol at pseudo steady state. *Psychopharmacology (Berl)*. 1993;110(4):471-8. doi:10.1007/BF02244655
5. Dikmen SS, Heaton RK, Grant I, Temkin NR. Test-retest reliability and practice effects of expanded Halstead-Reitan Neuropsychological Test Battery. *J Int Neuropsychol Soc*. May 1999;5(4):346-56.

4

Table S1. Schedule of assessment

	SCR		Study period 1 & 2					
			Day 1 & 22					Day 2-21
Time / Assessments	-60 days[4]	-1 h	0 h	1h	3h	4.5h	5h	
Informed consent	X							
Demography	X							
Inclusion and exclusion criteria	X							
Medical history	X							
Symptoms	X	X	X	X	X		X	
Arrival in clinic		X						
Explanation of NeuroCart and study procedures	X	X						
Vital signs (Weight, length, HR, BP & temp)		X						
Adverse events			X	X	X		X	X[7]
Questionnaires[1]		X						
Drug administration (oral)			X					X[8]
Cognitive test battery[3]		X		X	X		X	
Rinse mouth		X				X	X	
Saliva sample PK		X[6]				X	X	
Discharge							X	
trial@home device explanation to parents							X	
trial@home[2]								X
Teacher/caregiver questionnaire								X[5]

SCR = screening; PK = pharmacokinetic; HR = Heart rate; BP = Blood pressure

1. Questionnaires consist of: ABC questionnaire, and the Clinician's Global Impression (CGI-S at day 1, CGI-I at day 22).
2. Continuous physical activity, heart rate and sleep monitoring, as well as twice-weekly finger tapping, adaptive tracking and animal fluency.
3. NeuroCart tests consist of: animal fluency test, adaptive tracking, body sway, saccadic eye movements, smooth pursuit and tapping frequency.
4. Screening by phone.
5. ABC questionnaire on paper. Interview by phone for CGI-S (> day 1) and CGI-I (> day 21), conducted as soon as possible after the study-day.
6. Only on day 22
7. Evaluation adverse events by phone by study physician at Day 3 and Day 6 and when needed
8. Starting on the evening of Day 22, clonazepam will be tapered by decreasing the daily dose by 0.01 mg/kg/day every three days.

5

Table S2: *ARID1B* variants of included patients

ARID1B cDNA variant	*ARID1B* protein change	inheritance
NC_000006.11:g.(?_157144644)_(158028969_?)del	p.0	*de novo*
NM_020732.3:c.1146dup	p.(Gly383Argfs*152)	*not tested*
NM_020732.3:c.1160_1200del	p.(Ala387Glyfs*134)	*de novo*
NM_020732.3:c.1202del	p.(Gly401Alafs*29)	*de novo*
NM_020732.3:c.1320C>G	p.(Tyr440*)	*de novo*
NM_020732.3:c.1579C>T	p.(Gln527*)	*de novo*
NM_020732.3:c.2318C>G	p.(Ser773*)	*de novo*
NC_000006.11:g.157431700G>A	r.(spl?)	*not tested*
NM_020732.3:c.2519dup	p.(Tyr840*)	*de novo*
NM_020732.3:c.2917dup	p.(Met973Asnfs*16)	*not tested*
NM_020732.3:c.3478G>T	p.(Glu1160*)	*not tested*
NM_020732.3:c.4623_4641dup	p.(Asn1548Aspfs*94	*de novo*
NM_020732.3:c.4870C>T	p.(Arg1624*)	*de novo*
NM_020732.3:c.5072del	p.(Leu1691Argfs*75)	*de novo*
NM_020732.3:c.5404C>T	p.(Arg1802*)	*de novo*
NM_020732.3:c.5508del	p.(Ser1836Argfs*15)	*de novo*

6

Table S3: Clonazepam dosages per subject in drops of study medication*

Subject	201	202	203	204	205	206	207	208	209	210	211	212	213	214	215	216
**Weight (kg)**	65	60,5	57,1	55,4	24	51	28	39	50	36	38,6	68,5	19,1	55	74	54
**Dag 1**	4	3	3	3	1	3	2	2	3	2	2	4	1	3	4	3
**Dag 2**	4	3	3	3	1	3	2	2	3	2	2	4	1	3	4	3
**Dag 3**	4	3	3	3	1	3	2	2	3	2	2	4	1	3	4	3
**Dag 4**	6	6	6	3	2	6	3	4	6	4	2	6	1	6	6	6
**Dag 5**	6	6	6	3	2	6		4	6	4	2	6	2	6	6	6
**Dag 6**	6	6	6	3	2	6		4	6	2	2	6	2	6	6	6
**Dag 7**	6	6	6	6	4	6		6	6	2	1	3	3	6	4	6
**Dag 8**	6	6	3	6	4	6		6	6	2	1	3	3	6	4	6
**Dag 9**	6	6	3	6	4	6		6	6	2	1	3	3	6	4	6
**Dag 10**	6	6	3	6	4	6		6	6	2	1	3	3	6	4	6
**Dag 11**	6	6	6	6	4	6		6	6	2	2	3	3	6	4	6
**Dag 12**	6	6	6	6	4	6		6	6	2	2	3	3	6	4	6
**Dag 13**	6	6	6	6	4	6		6	6	2	2	3	3	6	4	6
**Dag 14**	6	6	6	6	4	6		6	6	2	2	3	3	6	4	6
**Dag 15**	6	6	6	6	4	6		6	6	2	2	M	3	6	4	6
**Dag 16**	6	6	6	6	4	6		6	6	2	2	3	3	6	4	6
**Dag 17**	6	6	6	6	4	6		6	6	2	2	3	3	6	4	6
**Dag 18**	6	6	6	6	4	6		6	6	2	2	3	3	6	4	6
**Dag 19**	6	6	6	6	4	6		6	6	2	2	3	3	6	4	6
**Dag 20**	6	6	6	6	4	6		6	6	2	2	3	3	6	4	M
**Dag 21**	6	6	6	6	4	6		6	6	2	M	3	3	6	4	M
**Dag 22**	6	6	6	6	4	6		6	6	2	2	3	3	6	4	6
**Dag 23**	4	3	3	3	2	3		2	3	0	1	1	1	3	2	3
**Dag 24**	4	3	3	3	2	3		2	3	0	1	1	1	3	2	3
**Dag 25**	4	3	3	3	2	3		2	3	0	1	1	1	3	2	3

* 1 drop = 0,083mg

7

Table S4: Self-reported improvements reported during CGI-I interview

Reported improvements	Clonazepam *n*=7	Placebo *n*=2
Expressive speech	4	1
Mental calmness	3	
Emotional outbursts/more susceptible to reason	2	
Concentration	2	1
Taking initiative	1	2
Indicating boundaries	1	
Sleep	5	

8

Table S5: Example of a SMART-defined goal using Goal Attainment Scaling

Goal attainment level	Score	SMART Goal X
Less than baseline	-3	[name] can concentrate on schoolwork for less than 3 consecutive minutes.
Baseline	-2	[name] can concentrate on schoolwork for about 3 minutes straight.
Goal partially achieved	-1	[name] can concentrate on schoolwork for 3-5 consecutive minutes.
Desired outcome	0	[name] can concentrate on her schoolwork for 5-10 consecutive minutes.
Somewhat better than desired outcome	1	[name] can concentrate on her schoolwork for 10-12 consecutive minutes.
Much better than desired outcome	2	[name] can concentrate on her schoolwork for >12 consecutive minutes.
Weight *(1=important; 2=very important; 3=most important)*	3	Most important
Probability of goal attainment *(1=doubtful; 2=possible; 3=probable)*	2	Possible

9

Table S6: N-of-1: Patient characteristics

	Sex	doses RCT*	doses N-of-1*
Subject 1	Female	2 daily 3 drops	2 daily 4 drops
Subject 2	Male	2 daily 6 drops	2 daily 3 drops
Subject 3	Female	2 daily 6 drops	2 daily 4 drops

* 1 drop = 0,083mg

10

Table S7: Treatment goals (ICF)

ICF level goals	Total number of goals*	Placebo<br>Number of (partially) achieved goals**	Blinded period/C<br>Number of (partially) achieved goals**	Clonazepam<br>Number of (partially) achieved goals**
Body functions and structures				
Sleep	2	1	2	2
Energy level	1	0	0	1
Concentration	2	0	1	2
Reaction time	1	0	1	0
Mental calmness***	1	NA	NA	NA
Activities and participation				
Communication/talking	2	0	1	1
Emotional fits	1	0	1	1
Social contact	1	1	0	1
Personal factors				
Dealing with anxiety	1	0	1	1

ICF: International Classification of Functioning, Disability and Health; NA=not applicable

* Subgoals were excluded

** average over period

*** not possible to evaluate

11

Table S8: Primary outcome

Subject		Placebo	Blinded period - clonazepam	Clonazepam	*p*-value*	*p*-value*
	GAS-T baseline	Average GAS-T	Average GAS-T	Average GAS-T	Placebo vs clonazepam (total study period)	Placebo vs clonazepam (blinded-period only)
Subject 1	23,5	24,5	27,3	32,2	0.06	0.19
Subject 2	19,4	23,0	31,2	35,9	0.04	0.18
Subject 3	19,4	21,5	51,3	56,4	0.03	<0.01
Group analysis					<0.01	<0.01

* *p*-value of GAS-T change from baseline

12

Table S9: Behavioral questionnaires in the *N*=1 study

Outcomes	Baseline		Placebo		Double-blind clonazepam		Clonazepam			
	Average	SD	Average	SD	Average	SD	Average	SD	*p*-value*	
<u>ABC</u>	*n*=3									
1: Irritability	6,00	8,72	6,06	9,08	6,33	8,50	0,83	1,04	0,05	a
2: Lethargy	14,67	9,45	14,50	9,26	16,50	12,50	8,58	7,47	0,11	a
3: Stereotypy	1,33	1,53	0,83	3,21	0,67	1,15	0,17	0,29	0,07	a
4: Hyperactivity	11,67	5,69	9,94	5,76	12,00	5,41	7,33	2,84	0,14	a
5: Inappropriate Speech	2,33	3,21	2,17	2,93	2,00	2,65	0,08	0,14	0,06	a
<u>CBCL</u>	*n*=2**									
Subscales										
1: Anxious/depressed	4,00	4,24	1,83	1,18	2,25	1,77	1,50	0,71	0,46	a
2: Withdrawn/depressed	6,00	2,83	6,00	3,30	6,25	3,18	6,00	3,54	0,93	a
3: Somatic complaints	1,00	1,41	1,50	0,71	2	0,00	1,00	1,41	0,83	a
4: Social problems	4,00	1,41	4,00	1,41	3,5	2,12	3,75	1,77	0,33	a
5: Thought problems	5,50	2,12	5,50	2,12	3,75	1,06	2,75	1,06	0,02	a
6: Attention problems	8,50	0,71	8,83	1,18	8,25	1,06	7,50	0,71	0,13	a
7: Rule-breaking behavior	0,50	0,71	0,67	0,94	1,5	2,12	1,00	1,41	0,58	a
8: Aggressive behavior	1,00	1,41	2,83	4,01	2	2,83	1,00	1,41	0,25	a
9: Other problems	2,50	0,71	3,50	1,18	4,5	3,54	3,75	2,47	0,60	a
Internalizing	11,00	0,00	9,33	2,83	10,50	1,41	8,50	4,24	0,47	a
Externalizing	1,50	2,12	3,50	4,95	3,50	4,95	2,00	2,83	0,49	a
1: Affective problems	3,50	0,71	3,00	1,89	3,50	0,00	2,00	0,71	0,26	a
2: Anxiety problems	3,00	1,41	1,67	0,47	3,00	1,41	1,75	0,35	0,89	a
3: Somatic problems	1,00	1,41	1,00	1,41	1,00	1,41	1,00	1,41	n.a.	a
4: Attention deficit/Hyperactivity problems	4,00	1,41	4,67	2,36	4,75	0,35	4,75	1,06	0,48	a
5: Oppositional/Defiant problems	4,00	1,41	4,67	2,36	4,50	0,71	4,75	1,06	0,48	a
6: Conduct problems	0,50	0,71	0,67	0,94	1,50	2,12	1,00	1,41	0,58	a
1: Sluggish cognitive tempo	2,50	0,71	3,17	0,24	2,50	1,41	2,25	0,35	0,05	a
2: Obsessive-Compulsive problems	1,00	1,41	1,33	1,89	0,75	1,06	1,25	1,77	0,73	a
3: Post-traumatic stress problems	6,00	0,00	5,67	0,47	7,25	1,77	5,00	0,00	0,65	a

* *p*-value over the total study period and change from baseline

a Linear mixed model analysis

** baseline measurement of subject 1 is missing

13
